# Lifestyle trajectories of boys and girls during the transition to secondary school in British Columbia, Canada, before and during the COVID-19 pandemic

**DOI:** 10.1186/s12889-025-26112-7

**Published:** 2026-01-06

**Authors:** Louise C. Mâsse, Olivia De-Jongh González, Karen Sauve, Claire N. Tugault-Lafleur, Lucy LeMare, Patti-Jean Naylor

**Affiliations:** 1 School of Population and Public Health (SPPH), Faculty of Medicine, University of British Columbia (UBC) & BC Children’s Hospital Research Institute (BCCHR), F512A-4480 Oak Street, BC V6H 3V4 Canada; 2 Faculty of Medicine, SPPH, UBC & BCCHR, Vancouver, BC Canada; 3 School of Nutrition Sciences, Faculty of Health Sciences, University of Ottawa, Ottawa, ON Canada; 4 Faculty of Education, Simon Fraser University, Burnaby, BC Canada; 5 University of Victoria, Victoria, BC Canada

**Keywords:** Children, Health behaviors, Secondary school transition, Gender, COVID-19 pandemic

## Abstract

**Background:**

The transition to secondary school is a critical period for adolescents, marked by increased autonomy and substantial changes in their physical and social environments, which can negatively influence lifestyle habits. The COVID-19 pandemic also affected these routines, but it remains unclear how adolescents’ behaviors shifted as they moved to secondary school during the pandemic.

**Objectives:**

We examined changes in adolescents’ lifestyle behaviors (screen time, sleep, sedentary time, physical activity (PA), and diet) during the transition to secondary school and explored whether changes were moderated by the pandemic or child gender.

**Methods:**

A sample of 689 adolescents had their health behaviors measured via self-report, 24-hour dietary recalls, and accelerometry at two time points: the final year of elementary school (grade 7) and the first year of secondary school (grade 8). 42% of the sample completed all data collection before the pandemic, and 58% during the pandemic. Covariate-adjusted mixed-effects models assessed behavioral trajectories over time, including 3-way interactions between time, pandemic exposure, and gender.

**Results:**

The transition to secondary school was generally associated with increased sedentary time and screen time, and reduced light PA, MVPA, likelihood of meeting sleep recommendations, and fruit and vegetables intake. However, some effects varied significantly by gender and cohort. For example, girls consistently had lower odds of meeting sleep recommendations and engaged in less MVPA than boys, while the pandemic cohort showed decreases in sedentary time and screen use (but remained above pre-pandemic levels), and increases in weekday fruit and vegetables intake.

**Conclusions:**

The transition to secondary school was associated with less healthy lifestyles, with variations by gender and pandemic exposure. To develop targeted interventions that promote positive habits during this critical life stage, it is necessary to identify the mechanisms driving these changes, as they may also reflect other developmental or environmental influences coinciding with the transition. Importantly, interventions should also address behavioral changes that occurred during the pandemic, particularly increases in recreational screen time that may persist into adulthood.

## Introduction

Worldwide, the prevalence of overweight and obesity among children aged 5–19 years has increased from 8% in 1990 to 20% in 2022, affecting 390 million children by 2022 [1]. In Canada, the prevalence of overweight and obesity among 12–17-year-olds has gradually increased by 10.7% since 2005, with 30.1% of adolescents affected in 2022 [2]. These statistics are alarming, as overweight and obesity during childhood and adolescence typically persists into adulthood [3], increasing the risk of non-communicable diseases and premature mortality [4].

Adolescence is a critical window for intervention, as this period is characterized by significant shifts in lifestyle behaviors associated with obesity, such as screen time, sleep, physical activity (PA), and dietary habits [5–8]. Specifically, the percentage of children spending more than 2 h on screens in their leisure time more than doubles from 31% in children aged 5–11 to 69% in those aged 12–17 [6]. Alarmingly, only 28.1% of Canadian adolescents meet the screen time recommendation, with fewer boys compared to girls (23.6% versus 32.8%) meeting this recommendation [6]. Sleep duration also declines with age and, in Canada, the percentage of children meeting sleep recommendations drops from 81% at the ages of 10–11 to 70% at the ages of 16–17 [9]. Studies also indicate a substantial reduction in PA ranging from 60% to 75% between the ages of 9–15 [10–13]. Furthermore, in Canada, only 7% of children achieve the recommended 60 min of moderate-to-vigorous PA (MVPA) daily, with boys typically being more active than girls [13]. Lastly, suboptimal dietary behaviors are prevalent during adolescence [7–15], with one third of Canadian children not consuming at least five fruits and vegetables per day, and one quarter of their calories coming from “other” foods [17]. Additionally, soft drink consumption increases with age by 20% for boys and 6% for girls [7].

 Moreover, the transition to secondary school is a key period for adolescents, characterized by increased autonomy, changes in friendships, physical and social environments, parental expectations, and increased academic demands, all of which can influence lifestyle behaviors [16–18]. A systematic review of 34 studies found strong consistent associations between the transition and increased sedentary time, and moderate evidence for decreased consumption of fruit and vegetables [19]. For snacking, sugar-sweetened beverages, screen time, and PA, the results were inconclusive or insufficient and no study examined the change in sleep [19]. A review of six longitudinal studies examining the transition, found that changes in PA levels, including the direction and magnitude, depended on type of PA assessed (e.g., light PA, MVPA, sports, active transport), context or setting (in-school versus out-of-school), and measurement methods (self-report versus device-measured) [20]. These findings highlight a substantial gap in understanding how this pivotal transition affects children’s diet, PA, screen time, and sleep.

The restrictions linked to the COVID-19 pandemic have also impacted some of these lifestyle factors negatively [21–24]. During the pandemic, adolescents’ recreational screen time increased, their sleep patterns and duration were all negatively affected, and PA declined [21–23]. Consistent with pre-pandemic conditions, girls were less active than boys, and adolescents (aged 12–17 years) were less active than younger children (aged 5–11 years) [21–23]. Dietary changes have also been reported, though findings are mixed, with studies documenting both positive and negative impacts on dietary habits [24]. Specifically, children consumed more food overall, including snacks, French fries, fruits, vegetables, grains, legumes, and baked products, while their intake of fast food and soft drinks decreased [24].

This paper examined changes in children’s lifestyle behaviors (screen time, sleep, sedentary time, PA, and diet) during the transition from elementary to secondary school, and explored whether the pandemic conditions and/or child gender moderated these changes. It was hypothesized that these behaviors would deteriorate during the transition, aligning with previous research [17, 19, 20, 25, 26]. However, the moderation effects are exploratory; therefore, no a priori hypotheses were made.

## Methods

### Design

**Fig. 1:**
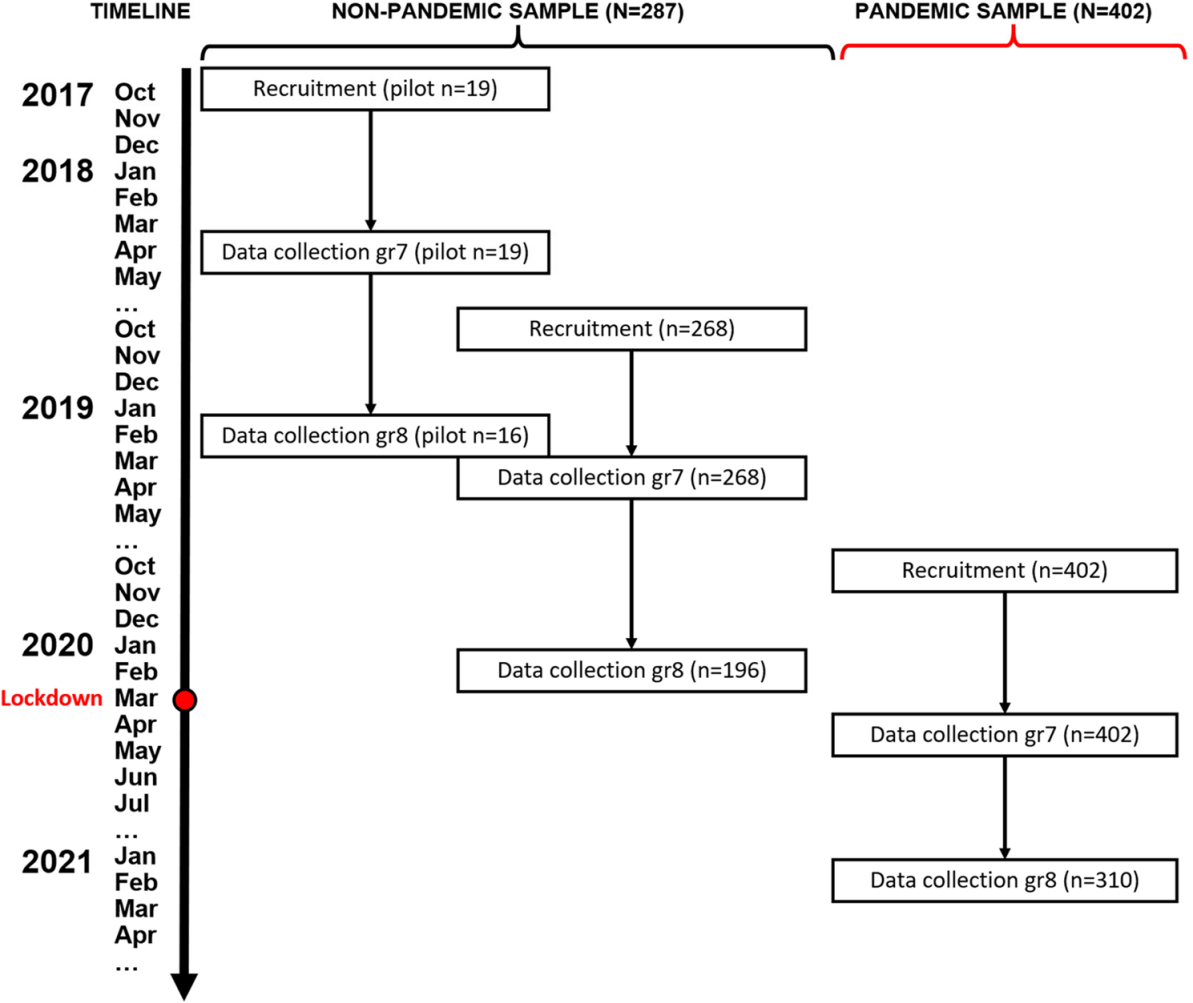
Timeline for family recruitment and data collection in pandemic and non-pandemic cohorts

This study analyzed data from the Health and Behaviors In Teens (HABITs) project, a prospective cohort study tracking children’s obesity-related outcomes from grade 7 to grade 8 during their transition to secondary school. Data were collected in waves from Spring 2018 to Spring 2021. A subset of participants experienced pandemic conditions, creating a naturalistic quasi-experimental design (see Fig. 1). Ethics approval was granted by the Children’s and Women’s Research Ethics Board at the University of British Columbia (H15-01876).

### Recruitment

Participants were recruited from public elementary schools in the Greater Vancouver area of British Columbia (BC), Canada, including districts in Vancouver, West Vancouver, Burnaby, Surrey, and Delta. These school districts were selected using non-probability convenience sampling while ensuring variation in school size and socioeconomic indicators based on the 2016 Canadian Census. Recruitment occurred over three years (2017–2019). For both the non-pandemic and pandemic cohorts, letters of introduction and consents were first sent to elementary school principals and principals of associated feeder secondary schools. Following district and school approval, grade 7 teachers were invited to facilitate in-class or virtual information sessions, during which students received study details and consent/assent forms for themselves and their parents. Informational packages were sent home with the students. Interested families enrolled in the study by returning completed forms to their children’s teachers or by providing consent online.

### Participants

The target sample included grade 7 students from schools where grade 7 was the final grade, which represents ~ 80% of BC schools. Students were eligible to participate if they: (1) were enrolled in a public elementary school in BC, (2) were able to read and write at a grade 6 level or above, and (3) had a parent or guardian who would also participate in the study. A total of 689 parent-child dyads enrolled in the study. Of these, 286 completed grade 7 data collection before the COVID-19 pandemic; 212 of them also completed grade 8 data collection and comprise the non-pandemic cohort. An additional 402 dyads completed grade 7 data collection during the pandemic, with 310 of them also completing grade 8 data collection, forming the pandemic cohort. All participants were nominally compensated for their involvement. Parents received $30 CAD and students received $55 CAD, with an additional opportunity to win $100 CAD through a draw if they completed all assessments and returned the accelerometer.

### Data collection procedures

For each dyad, data were collected at two time points: in grade 7 and in grade 8 (see Fig. 1). For the non-pandemic cohort, children’s grade 7 data were collected in schools, where they completed a 45-minute online questionnaire on iPads and were fitted with an Axivity AX3 wrist-worn accelerometer. Children were instructed to wear the AX3 on the non-dominant hand for eight days before returning them to their teachers. Children’s grade 8 data were collected outside of school hours, either at community centers or at the BC Children’s Hospital Research Institute, with the same assessments repeated. For children in the pandemic cohort, both grade 7 and 8 data collections were conducted remotely due to COVID-19-related restrictions. Children completed questionnaires online, and accelerometers were mailed to their homes along with prepaid return envelopes. The pandemic cohort did not wear accelerometers in grade 7, as the Ethics Board did not permit the research team to distribute devices during that period (spring of 2020). The procedures for parents were the same in both grades and cohorts, with questionnaires completed online.

### Exposure to pandemic conditions

In BC, schools initially closed on March 17, 2020, and students returned to in-person classes in June 2020. Schools remained open throughout the 2020/2021 academic year, except for scheduled winter and spring breaks. Grade 8 students returned to a reduced-time-in-school schedule, with 50% present either in the morning or afternoon (see Fig. 1). For the purpose of this study, pandemic exposure was a binary indicator where cohorts were categorized as ‘non-pandemic’ or ‘pandemic’.

### Measures

#### Self-reported

*Sociodemographic *data were collected using items adapted from the General Social Survey [27] and the Canadian Community Health Survey [28]. Adolescent gender (“*How do you describe yourself?*”) was a key moderator examined, and race/ethnicity (“*People living in Canada come from different cultural and racial backgrounds. Please read all the categories and select all that apply to your child*”) and household income (“*What is your best estimate of TOTAL income*,* before taxes and deductions*,* of ALL household members from all sources in the past 12 months?*”) were included as covariates.

*Dietary intake* was measured with the online 2016 Automated Self-Administered 24-hour (ASA24-Canada) Canadian Dietary Assessment Tool [29]. Data were collected over 3 days including a weekend. Students selected from 1100 food and beverage options and used photographs to estimate quantities. Data were processed using the 2015 Canadian Nutrient data file, which provide estimates of four food groups (vegetables and fruit, grain products, milk and alternatives, and meat and alternatives) based on the 2007 Canada’s Food Guide. A diet quality index was computed with the 2015 U.S. Healthy Eating Index (HEI) SAS macro [30] adapted to the 2007 Canadian recommendations with scores ranging from 0 to 100. HEI scores can be interpreted as follows: poor (≤ 50), needs improvement (50–80), good (≥ 80) [31]. Key variables examined in this study include servings of fruits and vegetables and overall diet quality represented by the HEI score.

*MVPA* was measured using the modified PA Questionnaire for Children (PAQ-C) [32], which shows high test re-test reliability (*r* = 0.75 to 0.82) and adequate validity against accelerometry (*r* = 0.34) [32]. Children reported how many days per week in the past week they were physically active (had their heart rate increased and were out of breath) for at least 60 min. Response options ranged from 0 to 7 days.

*Screen time* was self-reported using the Take Action question [33], which has high test re-test reliability (*r* = 0.51 to 0.93) and moderate correlation with the International PA Questionnaire (*r* = 0.24 to 0.37) [34, 35]. Children reported on previous weekday: (a) Recreational screen time such as using a computer, tablet, or mobile device outside of schoolwork, and (b) Screen-watching time such as watching TV, movies, sports. Response options for both questions ranged from 0 min to 4 + hours (coded as 240 min).

*Sleep* was assessed by asking children about their wake-up and bedtime on a regular weekday and weekend day [36]. Hours of sleep were then calculated and compared to Canadian recommendations (9–11 h for 5–13-year-olds and 8–10 h for 14–17-year-olds [37]).

#### Accelerometry

*Movement behaviors (light PA*,* MVPA*,* sedentary time*,* and sleep)* were evaluated using Axivity AX3 accelerometers (Axivity Ltd., Newcastle, UK) worn on the non-dominant wrist for eight consecutive days in both grade 7 and 8, except for participants in the pandemic cohort, who worn the devices in grade 8 only. The devices recorded raw triaxial acceleration data at 100 Hz (i.e., 100 data points per second) within a seismic acceleration range of ± 8 g, where g corresponds to the Earth’s gravitational pull, across three axes: vertical, anteroposterior, and mediolateral. Raw acceleration signals were processed in three steps: First, three independent reviewers used OmGui software (Open Movement & Newcastle University, UK), alongside participants’ activity diaries and study schedules, to visualize raw data and identify ‘invalid’ periods at the start and end of data collection when devices were being transported by research staff and not worn. These transport periods were then deleted from the dataset, which resulted in partial data on days 1 and 8. A Python script was used to merge valid portions of data from day 1 (afternoon) and day 8 (morning), which occurred on the same day of the week. This dataset was processed in R using the GGIR package [38], which implements the van Hees et al. algorithm [39, 40] for automatic calibration and non-wear detection. Non-wear periods were defined as intervals with a standard deviation < 13 mg and range < 50 mg per axis over 15-minute windows, with a minimum duration ≥ 60 min. This approach reduces sensitivity to spurious movements when the device is not worn. The algorithm also identifies sustained abnormal values and calculates average dynamic acceleration. PA intensities were classified using acceleration thresholds integrated in GGIR [41] for children aged 8–14 years (aligning with the age of our study’s child sample): light (≥ 87.5 mg) and moderate-to-vigorous (≥ 250 mg). These thresholds account for wearing the accelerometer on the non-dominant hand and are based on Phillips’ calibration [42], which validated the cut-points for the GENEActiv accelerometer (Activinsight Ltd, Cambs, UK) in children aged 8–14. Acceleration values and cut-points from this device have been shown to be equivalent to those of the Axivity AX3 device used in this study [43]. GGIR also includes validated methods for sleep detection using wrist-worn accelerometer data, with or without input from a sleep diary [38]. In this study, sleep was estimated using GGIR’s sleep detection algorithm, which identifies periods of sustained low movement and minimal changes in arm posture to determine sleep onset and wake time. The algorithm was guided by participant-reported sleep diaries, which improves the accuracy of sleep window identification. Sedentary time was classified as periods of minimal movement occurring outside the identified sleep window, with these sustained inactivity bouts interpreted as sedentary behavior. GGIR generates variables based on two timeframes: midnight-to-midnight (a 24-hour calendar day) and wake-up-to-wake-up (a variable-length day). For the purposes of this study, the midnight-to-midnight definition was used for all waking movement behaviors (sedentary time, light PA, and MVPA), while the wake-up-to-wake-up definition was used for sleep duration.

### Statistical analysis

Grade 7 demographic differences between cohorts were assessed with Student t test and Pearson Chi square (X^2^) test for continuous and categorical variables, respectively. Changes in behaviors were analyzed using multilevel mixed-effect logistic (sleep variables) and linear models (all remaining variables). For each behavior (PA, screen time, sedentary time, sleep, fruit and vegetables intake, and HEI score), we first evaluated the main effects of grade, cohort, and gender. Subsequently, we performed moderation analyses that included a three-way interaction (grade-by-cohort-by-gender), as well as all first and second order terms (i.e., two-way interactions and individual term effects). For device-measured PA, the three-way interactions could not be tested as grade 7 accelerometry data were not collected during the pandemic. Therefore, separate models were run to test: (1) grade-by-gender effect in the non-pandemic cohort using mixed-effect models, and (2) cohort-by-gender effect in grade 8 using linear regression models. All moderation effects were examined by plotting and decomposing the interactions into simple effects. All models controlled for demographic covariates and were analyzed using STATA version 15 [44]. Significance of main and simple effects was set at *p* ≤ 0.01, in addition to considering whether the differences were of practical public health significance. However, given the theoretical expectation of gender- and pandemic-related subgroup differences, we decomposed and plotted interactions with *p* < 0.10, consistent with recommendations from Aiken and colleagues [45]. This threshold aligns with guidance from Stone-Romero and Liakhovitski [46], who argue that moderation effects often suffer from low statistical power in applied observational research, and that a more lenient Type I error rate (defined as *p* < 0.10 or *p* < 0.15 in some cases) may be appropriate under such condition.

## Results

### Descriptive data on sociodemographic characteristics and lifestyle behaviors

 Table 1 shows similar socio-demographic characteristics between the non-pandemic and pandemic cohorts, with no significant differences between them at baseline, except for child age due to temporality, as the pandemic sample had data collected slightly later during the year. Child gender was nearly equally distributed between boys and girls, with most children (~ 87%) from two-parent households. Approximately 58% of parents had a university degree, ~ 31% reported household income under $70,000 CAD, and ~ 36% self-identified as white.

**Table 1:** Participants’ socio-demographic characteristics

		Non-pandemic cohort *N* = 287				Pandemic cohort *N* = 402			
		Grade 7 *n* = 287		Grade 8 *n* = 212		Grade 7 *n* = 402		Grade 8 *n* = 310	
Socio-demographic characteristics		*n*	%	*n*	%	*n*	%	*n*	%
Children									
Age years (mean ± SD)		12.8	0.5	13.8	0.5	12.9	0.3	13.8	0.4
Gender (n, %)	Boys	138	48.3	98	46.2	179	46.0	143	46.4
	Girls	148	51.8	114	53.8	210	54.0	165	53.6
Parents/primary guardians									
Age years (mean ± SD)		46.3	± 5.1	47.0	± 5.0	46.1	± 5.5	47.1	± 5.4
Gender (n, %)	Fathers	76	27.4	55	25.9	80	21.7	68	22.5
	Mothers	201	72.6	157	74.1	289	78.3	234	77.5
Marital status (n, %)	Single, separated, divorced & widowed	39	14.3	30	14.3	44	12.1	29	9.8
	Married/Living Common-Law	233	85.7	180	85.7	319	87.9	267	90.2
Education(n, %)	Non-University degree	118	42.6	83	39.2	163	43.8	124	40.9
	Bachelor degree	101	36.5	81	38.2	118	31.7	100	33.0
	Master/doctorate/professional degree	58	20.9	48	22.6	91	24.5	79	26.1
Household income (n, %)	Less than $70,000CAD	80	31.9	62	31.5	98	30.0	83	30.0
	$70,000 - $99,999CAD	57	22.7	43	21.8	61	18.7	50	18.1
	$100,000 - $124,999CAD	39	15.5	32	16.2	41	12.5	34	12.3
	$125,000 - $149,999CAD	25	10.0	19	9.6	31	9.5	29	10.5
	$150,000CAD or higher	50	19.9	41	20.8	96	29.4	81	29.2
Race/ethnicity (n, %)	White	105	37.9	83	39.2	130	35.3	114	37.8
	East and South East Asian	93	33.6	71	33.5	126	34.2	107	35.4
	South Asian	43	15.5	34	16.0	57	15.5	40	13.3
	Others & mixed	36	13.0	24	11.3	55	15.0	41	13.6

Percentages may not add up to 100 due to rounding

*N* = 689

Table 2 shows lifestyle behaviors among children in both cohorts. Children in the pandemic cohort had significantly greater consumption of fruit and vegetables, and higher recreational screen time and screen-watching time during weekdays.

**Table 2:** Descriptive statistics for children’s lifestyle behaviors

	Non-pandemic cohort *N* = 287				Pandemic cohort *N* = 402			
	Grade 7 *n* = 287		Grade 8 *n* = 212		Grade 7 *n* = 402		Grade 8 *n* = 310	
Children’s lifestyle behaviors	Mean	±SD	Mean	±SD	Mean	±SD	Mean	±SD
	**or %**		**or %**		**or %**		**or %**	
*Self-reported*								
HEI Total (weekday)	54.3	± 10.0	53.3	± 10.9	54.5	± 10.8	53.6	± 11.1
HEI Total (weekend)	52.2	± 11.9	52.1	± 13.9	51.9	± 11.8	53.0	± 13.2
Fruit & vegetables (cup eq./weekday)*	**1.4**	± 1.0	1.3	± 1.2	**1.9**	± 2.3	1.4	± 1.2
Fruit & vegetables (cup eq./weekend day)	1.4	± 1.3	1.2	± 1.0	1.7	± 1.5	1.3	± 1.4
Recreational screen time (hours/weekday)*	**87.6**	± 67.6	124.4	± 77.0	**169.6**	± 76.8	148.9	± 83.3
Screen watching time (hours/weekday)*	**62.0**	± 60.0	72.7	± 73.8	**96.9**	± 80.1	144.3	± 83.0
MVPA (# days/week with 60 + min)	3.8	± 1.8	3.5	± 1.9	3.9	± 2.3	3.5	± 2.2
Sleep (meeting guidelines on weekdays)	65.8%		56.7%		68.0%		56.2%	
Sleep (meeting guidelines on weekend days)	63.8%		60.6%		67.3%		57.2%	
*Accelerometry*								
MVPA (min/weekday)	80.2	± 31.9	64.9	± 29.2	--	--	62.8	± 29.9
MVPA (min/weekend day)	52.3	± 32.4	44.1	± 32.8	--	--	45.2	± 33.5
Light PA (min/weekday)	204.7	± 49.6	174.5	± 45.1	--	--	173.6	± 68.8
Light PA (min/weekend day)	177.8	± 67.0	145.8	± 57.5	--	--	156.0	± 65.5
Sedentary time (hrs/weekday)	11.5	± 1.6	12.4	± 1.1	--	--	11.9	± 1.6
Sedentary time (hrs/weekend day)	11.6	± 1.5	11.9	± 1.7	--	--	11.4	± 1.7
Sleep (meeting guidelines on weekdays)	22.2%		12.1%		--	--	28.1%	
Sleep (meeting guidelines on weekend days)	27.6%		19.4%		--	--	38.2%	

Star (*) and bolded values indicate significant (p≤0.01) differences between cohorts at baseline (grade 7)

*HEI* Healthy Eating Index, *PA* Physical activity, *MVPA* Moderate-to-vigorous PA, *SD* Standard deviation

*N* = 689

-- = Not assessed

### Associations of school transition, pandemic exposure, and gender, with self-reported lifestyle behaviors

**Fig. 2:**
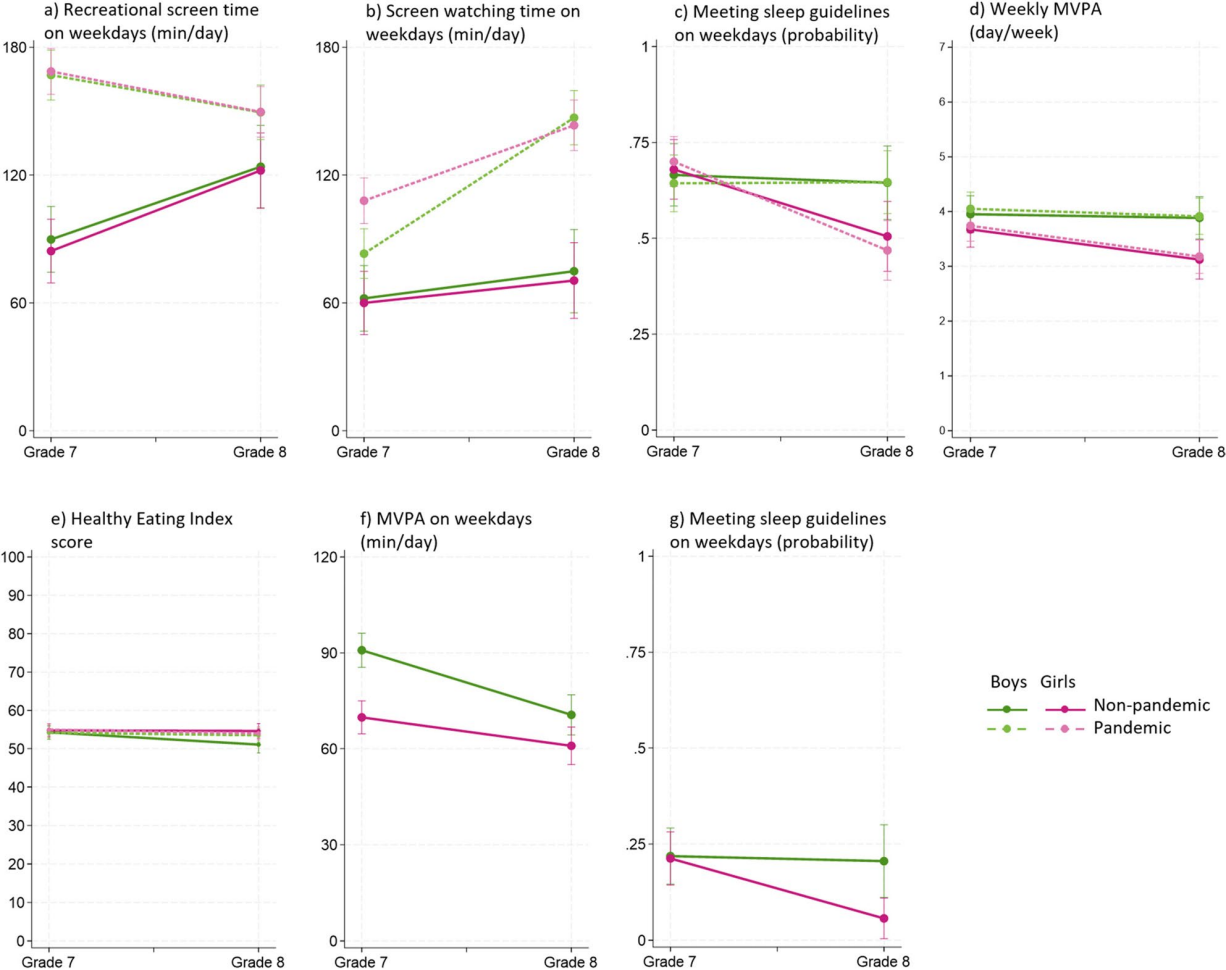
Interaction between school transition, pandemic, and gender, on self-reported (**a**-**e**) and accelerometry-measured (**f**-**g**) lifestyle behaviors

Tables 3 and 4 show changes in children’s self-reported lifestyle behaviors. The analysis also examined whether pandemic exposure and/or children’s gender moderated these behavioral changes, with meaningful effects depicted in Fig. 2 (a-e).

**Table 3:** Effects of school transition, pandemic, and gender on self-reported movement behaviors

		Recreational screen time (min/weekday)			Screen-watching time (min/weekday)			Meeting sleep guidelines on weekdays (vs. not)			Meeting sleep guidelines on weekend (vs. not)			60 + min MVPA (# days/week)		
		b	*p*	95%CI	b	*p*	95%CI	OR	*p*	95%CI	OR	*p*	95%CI	b	*p*	95%CI
Grade main effect		−2	0.720	−10;7	**37**	**0.000**	**28;46**	**0.57**	**0.000**	**0.43;0.75**	**0.64**	**0.004**	**0.47;0.87**	**−0.4**	**0.000**	**−0.5;−0.2**
Cohort main effect		**59**	**0.000**	**48;71**	**51**	**0.000**	**40;61**	0.97	0.818	0.72;1.29	1.02	0.918	0.70;1.49	0.1	0.615	−0.2;0.3
Gender main effect		0	0.961	−11;11	7	0.198	−4;17	0.78	0.099	0.59;1.05	0.89	0.552	0.62;1.30	**−0.5**	**0.000**	**−0.8;−0.2**
Grade-by-gender interaction		4	0.813	−27;34	−2	0.891	−35;30	**0.48**	**0.078**	**0.21;1.09**	1.06	0.901	0.43;2.62	**−0.5**	**0.096**	**−1.1;0.1**
Cohort-by-gender interaction		7	0.602	−20;34	**27**	**0.049**	**0;54**	1.25	0.583	0.57;2.73	1.03	0.951	0.40;2.63	0.0	0.920	−0.7;0.6
Grade-by-cohort interaction		**−52**	**0.000**	**−79;−25**	**51**	**0.000**	**23;80**	1.13	0.772	0.50;2.52	0.71	0.456	0.30;1.73	−0.1	0.808	−0.8;0.7
Grade-by-cohort-by-gender interaction		−5	0.782	−42;31	−26	0.188	−65;13	0.67	0.477	0.23;2.01	0.82	0.739	0.25;2.71	0.1	0.880	−0.7;0.8
Decomposed interaction (simple) effects																
Grade (Gr)	Gr8 (vs. 7) in non-pandemic boys	**34**	**0.003**	**12;57**	13	0.291	−11;37	0.90	0.738	0.49;1.66	0.80	0.505	0.41;1.55	−0.1	0.748	−0.5;0.3
	Gr8 (vs. 7) in pandemic boys	−18	0.020	−32;−3	**64**	**0.000**	**48;80**	1.02	0.955	0.60;1.72	0.57	0.062	0.32;1.03	−0.1	0.458	−0.5;0.2
	Gr8 (vs. 7) in non-pandemic girls	**38**	**0.000**	**17;58**	11	0.347	−11;32	**0.43**	**0.004**	**0.24;0.76**	0.85	0.594	0.46;1.57	**−0.6**	**0.006**	**−0.9;−0.2**
	Gr8 (vs. 7) in pandemic girls	**−19**	**0.007**	**−33;−5**	**35**	**0.000**	**21;50**	**0.32**	**0.000**	**0.20;0.54**	**0.49**	**0.010**	**0.29;0.84**	**−0.6**	**0.001**	**−0.9;−0.2**
Cohort	Pandemic (vs. non-pandemic) in Gr7 boys	**77**	**0.000**	**58;97**	21	0.034	2;40	0.89	0.698	0.51;1.57	1.23	0.553	0.62;2.43	0.1	0.674	−0.4;0.6
	Pandemic (vs. non-pandemic) in Gr8 boys	26	0.032	2;49	**72**	**0.000**	**49;96**	1.01	0.981	0.53;1.90	0.88	0.734	0.42;1.86	0.0	0.908	−0.5;0.5
	Pandemic (vs. non-pandemic) in Gr7 girls	**84**	**0.000**	**66;103**	**48**	**0.000**	**30;66**	1.11	0.696	0.65;1.92	1.27	0.472	0.67;2.41	0.1	0.764	−0.4;0.5
	Pandemic (vs. non-pandemic) in Gr8 girls	28	0.012	6;49	**73**	**0.000**	**51;94**	0.84	0.553	0.48;1.48	0.74	0.382	0.37;1.46	0.1	0.817	−0.4;0.5
Gender	Girls (vs. boys) in non-pandemic Gr7	−6	0.618	−27;16	−2	0.846	−24;19	1.08	0.803	0.60;1.94	0.90	0.762	0.45;1.81	−0.3	0.245	−0.7;0.2
	Girls (vs. boys) in pandemic Gr7	2	0.835	−14;18	**25**	**0.002**	**9;41**	1.34	0.261	0.80;2.24	0.92	0.803	0.50;1.72	−0.3	0.143	−0.7;0.1
	Girls (vs. boys) in non-pandemic Gr8	−2	0.894	−28;25	−4	0.746	−31;22	0.51	0.042	0.27;0.98	0.95	0.897	0.44;2.05	**−0.8**	**0.005**	**−1.3;−0.2**
	Girls (vs. boys) in pandemic Gr8	0	0.981	−17;18	−4	0.690	−21;14	**0.43**	**0.003**	**0.25;0.75**	0.80	0.503	0.41;1.54	**−0.7**	**0.001**	**−1.2;−0.3**

Reference groups: not meeting sleep recommendations, boys, grade 7, and pandemic cohort. Bolded values indicate *p*≤0.01 (main and simple effects) or *p* < 0.1 (interaction effects)

*MVPA* Moderate-to-vigorous physical activity, *b* unstandardized coefficient from mixed-effect linear model, *OR* odd ratios from logistic mixed-effect models

**Table 4:** Effects of school transition, pandemic, and gender on self-reported dietary behaviors

		HEI on weekdays (%)			HEI on weekend (%)			Fruit & Vegetables on weekdays (cup eq.)			Fruit & Vegetables on weekend (cup eq.)		
		b	*p*	95%CI	b	*p*	95%CI	b	*p*	95%CI	b	*p*	95%CI
Grade main effect		−1.1	0.033	−2.2;−0.1	0.5	0.627	−1.5;2.5	**−0.26**	**0.001**	**−0.43;−0.11**	**−0.28**	**0.008**	**−0.49;−0.07**
Cohort main effect		0.3	0.658	−1.1;1.8	0.1	0.940	−2.1;2.3	**0.34**	**0.002**	**0.12;0.55**	0.21	0.078	−0.02;0.45
Gender main effect		1.1	0.126	−0.3;2.6	1.4	0.225	−0.8;3.5	0.22	0.039	0.01;0.44	0.13	0.261	−0.10;0.37
Grade-by-gender		3.1	0.055	−0.1;6.2	1.6	0.576	−3.9;7.1	0.04	0.879	−0.44;0.52	0.33	0.267	−0.25;0.90
Cohort-by-gender		0.3	0.862	−3.1;3.7	−0.9	0.750	−6.7;4.8	−0.07	0.797	−0.58;0.45	−0.06	0.842	−0.67;0.55
Grade-by-cohort		2.5	0.107	−0.6;5.6	0.6	0.843	−5.3;6.5	−0.31	0.202	−0.78;0.17	0.02	0.957	−0.60;0.64
Grade-by-cohort-by-gender interaction		−3.4	0.115	−7.6;0.8	0.6	0.878	−7.4;8.7	−0.09	0.774	−0.74;0.55	−0.20	0.639	−1.04;0.64
Decomposed interaction (simple) effects													
Grade (Gr)	Gr8 (vs. 7) in non-pandemic boys	**−3.2**	**0.007**	**−5.6;−0.9**	−0.8	0.707	−4.9;3.3	−0.09	0.625	−0.45;0.27	−0.42	0.055	−0.85;0.01
	Gr8 (vs. 7) in pandemic boys	−0.7	0.508	−2.7;1.3	−0.2	0.930	−4.5;4.1	−0.40	0.012	−0.71;−0.09	−0.40	0.077	−0.85;0.04
	Gr8 (vs. 7) in non-pandemic girls	−0.1	0.894	−2.2;2.0	0.8	0.674	−2.9;4.4	−0.05	0.752	−0.37;0.27	−0.10	0.624	−0.48;0.29
	Gr8 (vs. 7) in pandemic girls	−1.0	0.318	−2.9;0.9	2.0	0.334	−2.1;6.1	**−0.45**	**0.002**	**−0.75;−0.16**	−0.28	0.201	−0.71;0.15
Cohort	Pandemic (vs. non-pandemic) in Gr7 boys	−0.1	0.936	−2.6;2.4	0.2	0.938	−4.1;4.4	**0.52**	**0.006**	**0.15;0.90**	0.29	0.206	−0.16;0.74
	Pandemic (vs. non-pandemic) in Gr8 boys	2.4	0.091	−0.4;5.3	0.8	0.741	−3.8;5.3	0.22	0.318	−0.21;0.64	0.31	0.211	−0.17;0.79
	Pandemic (vs. non-pandemic) in Gr7 girls	0.2	0.867	−2.1;2.5	−0.8	0.701	−4.7;3.2	0.46	0.011	0.11;0.81	0.23	0.282	−0.19;0.65
	Pandemic (vs. non-pandemic) in Gr8 girls	−0.6	0.627	−3.2;1.9	0.5	0.831	−3.7;4.6	0.05	0.782	−0.33;0.44	0.04	0.884	−0.40;0.49
Gender	Girls (vs. boys) in non-pandemic Gr7	0.5	0.704	−2.0;3.0	0.9	0.633	−2.9;4.7	0.27	0.165	−0.11;0.64	0.06	0.769	−0.34;0.46
	Girls (vs. boys) in pandemic Gr7	0.8	0.500	−1.5;3.1	0.0	0.993	−4.4;4.3	0.20	0.257	−0.15;0.54	0.00	0.993	−0.46;0.46
	Girls (vs. boys) in non-pandemic Gr8	3.6	0.015	0.7;6.4	2.5	0.265	−1.9;6.9	0.30	0.168	−0.13;0.74	0.39	0.103	−0.08;0.85
	Girls (vs. boys) in pandemic Gr8	0.5	0.695	−2.0;3.0	2.2	0.324	−2.2;6.5	0.14	0.453	−0.23;0.51	0.12	0.601	−0.34;0.58

Reference groups: not meeting sleep recommendations, boys, grade 7, and pandemic cohort. HEI = Healthy Eating Index – Canada; cup eq. = cup equivalent; b = unstandardized coefficient from mixed-effect linear model, OR = odd ratios from logistic mixed-effect models. Bolded values indicate *p*≤0.01 (main and simple effects) or *p* < 0.1 (interaction effects)

*Recreational screen time* on weekdays showed a significant grade-by-cohort interaction, which is explained by the simple effects (Table 3, Fig. 2a). In the non-pandemic cohort, the transition led to daily increases of 34–38 min in weekday recreational screen time, while in the pandemic cohort, it significantly decreased by ~ 19 min per day. Despite these opposing trends, the pandemic cohort had higher overall recreational screen time in particular in grade 7, with 77–84 more minutes per day.

*Screen-watching time* on weekdays showed significant cohort-by-grade and cohort-by-gender interactions (Table 3, Fig. 2b). In the non-pandemic cohort, weekday screen-watching time remained relatively stable with the transition regardless of gender. However, in the pandemic cohort, it increased sharply with the transition, and more so among boys compared to girls (64 versus 35 min per day), even though grade 7 girls had reported significantly higher levels of screen time than grade 7 boys (specifically, 25 more minutes per weekday). Overall screen-watching time was significantly higher during the pandemic, with greater differences in grade 8 (72–73 min per day) relative to grade 7 (21–48 min per day).

*Self-reported sleep* on weekdays showed a significant grade-by-gender interaction, as the odds of meeting sleep recommendations significantly decreased for girls in both cohorts (non-pandemic OR = 0.43, pandemic OR = 0.32) as opposed to boys, for whom weekday sleep did not significantly change (Table 3, Fig. 2c). Conversely, weekend day sleep did not show subgroup differences, but only a main effect associated with the school transition. Specifically, the odds of meeting sleep guidelines on weekends decreased (OR = 0.57) in the averaged effects of grade across levels of the moderator variables.

*Self-reported MVPA* showed a significant grade-by-gender interaction (Table 3, Fig. 2d). Specifically, girls’ MVPA participation in both cohorts decreased significantly by about half day per week post-transition, as opposed to boys’ MVPA which did not significantly change. This resulted in meaningful differences of about ¾ (0.7–0.8) of a day per week between boys and girls in grade 8.

*HEI* on weekdays (Table 4, Fig. 2e) showed a significant grade-by-gender interaction; however, this was deemed not meaningful, as it reflected a difference of only 3.1% points. Given that the standard deviation for HEI ranges from 10% to 11%, this small change (approximately 1/3 of a standard deviation) is unlikely to be of public health significance. On weekends, no significant associations emerged for HEI.

*Fruit and vegetables consumption* on weekdays and weekend days did not show subgroup differences but only effects at the averaged level (i.e., main effects). Specifically, significant grade effects indicated that consumption significantly decreased by about ¼ of cup per day (weekdays b=−0.26, weekend days b=−0.28, see Table 4). In addition, weekday consumption had a significant cohort effect, which indicates that fruit and vegetables intake was higher by ⅓ of a cup per day in the pandemic cohort as compared to the non-pandemic cohort.

### Associations of school transition, pandemic exposure, and gender, with accelerometer-measured lifestyle behaviors

Tables 5 and 6 show changes in children’s device-measured movement behaviors (light PA, MVPA, sedentary time, and sleep) and relevant interaction effects are shown in Fig. 2 (f-g). Accelerometry-measured movement behaviors were analyzed separately for the grade and cohort effects as accelerometry data were unavailable for grade 7 during the pandemic.

*Accelerometry-measured light PA* did not show subgroup differences, as no significant interactions emerged. Only main effects emerged for grade in model 1 for both weekdays and weekend days (non-pandemic cohort only, as accelerometry data is not available for the pandemic cohort in grade 7). Specifically, light PA significantly declined from grade 7 to 8 by approximately 30–34 min per day, regardless of gender (Table 5). Model 2, which compared non-pandemic and pandemic cohorts at grade 8, found no significant effects on light PA.

*Accelerometry-measured MVPA* on weekdays showed a significant grade-by-gender interaction in the non-pandemic cohort. While the transition to secondary school significantly reduced weekday MVPA for both genders, boys experienced a greater decline compared to girls (declines of 20 versus 9 min per weekday, respectively). While girls consistently had lower MVPA than boys, the gender gap narrowed in grade 8 compared to a clear 21-minute difference observed in grade 7 (Table 5 and Fig. 2f). On weekends, no significant grade-by-gender interaction was observed. Instead, main effects indicated that the transition to secondary school and being a girl were each significantly associated with declines in MVPA. Specifically, the main effects showed that MVPA decreased by 8 min per day following the transition, and that, across both grades, girls engaged in 18 fewer minutes of MVPA per day than boys. Model 2, which compared non-pandemic and pandemic cohorts at grade 8, revealed no significant effects for weekday MVPA. On weekends, only a main effect of gender was observed; where girls engaged in approximately 10 fewer minutes of MVPA per day than boys.

*Accelerometry-measured sedentary time* on weekdays and weekend days showed significant main effects but not subgroup differences in Model 1 (non-pandemic cohort). Specifically, sedentary time increased by approximately 56 min (0.93 h*60) on weekdays and 25 min (0.41 h*60) on weekend days after the school transition across both gender levels measured (Table 6). Model 2 (comparing both cohorts at grade 8) showed a significant cohort effect for sedentary time only on weekdays, with the pandemic cohort accumulating approximately 32 fewer minutes per day than the non-pandemic cohort (0.53 h*60).

*Accelerometry-measured sleep* showed a significant grade-by-gender interaction in Model 1 (non-pandemic cohort). Specifically, girls had significantly lower odds of meeting weekday sleep recommendations after the transition to secondary school (OR = 0.19), whereas boys’ odds did not change significantly, despite boys and girls having similar proportions meeting sleep guidelines in grade 7 (Table 6, Fig. 2g). On weekends, no significant interactions or grade effects were observed. However, being a girl was associated with lower odds of meeting sleep recommendations across both grades (OR = 0.39). When comparing non-pandemic and pandemic cohorts in grade 8 (Model 2), no significant cohort-by-gender interactions emerged (Table 6). Instead, being in the pandemic cohort was associated with higher odds of meeting sleep guidelines on both weekdays and weekend days (OR = 2.89 and 2.39, respectively), and a main effect of gender indicated that girls had lower odds of meeting sleep recommendations on weekdays than boys (OR = 0.40).

**Table 5:** Effects of school transition, pandemic, and gender on accelerometry-measured physical activity

		Light PA (min/weekdays)			Light PA (min/weekend day)			MVPA (min/weekdays)			MVPA (min/weekend day)		
		b	*p*	95%CI	b	*p*	95%CI	b	*p*	95%CI	b	*p*	95%CI
Model 1: Mixed-effect linear regression models testing grade and gender main effects and their interaction in the non-pandemic cohort^a^													
Grade main effect		**−30**	**0.000**	**−37;−22**	**−34**	**0.000**	**−45;−23**	**−14**	**0.000**	**−19;−10**	**−8**	**0.004**	**−13;−3**
Gender main effect		−1	0.878	−12;10	1	0.931	−14;16	**−17**	**0.000**	**−23;−10**	**−18**	**0.000**	**−25;−10**
Grade-by-gender interaction		−8	0.315	−23;8	−2	0.851	−25;20	**11**	**0.013**	**2;20**	4	0.453	−6;14
Decomposed interaction (simple) effects													
Grade	Grade 8 (vs. 7) among boys	**−25**	**0.000**	**−37;−14**	**−32**	**0.000**	**−49;−16**	**−20**	**0.000**	**−27;−14**	−10	0.013	−18;−2
	Grade 8 (vs. 7) among girls	**−33**	**0.000**	**−44;−23**	**−35**	**0.000**	**−49;−20**	**−9**	**0.004**	**−15;−3**	−6	0.090	−13;1
Gender	Girls (vs. boys) in grade 7	2	0.726	−10;15	2	0.865	−16;19	**−21**	**0.000**	**−29;−14**	**−19**	**0.000**	**−28;−11**
	Girls (vs. boys) in grade 8	−6	0.436	−20;9	−1	0.951	−21;20	−10	0.027	−18;−1	**−15**	**0.002**	**−25;−6**
Model 2: Linear regression models testing cohort and gender main effects and their interaction in grade 8 students from both cohorts^a^													
Cohort main effect		−1	0.853	−13;11	8	0.265	−6;22	−2	0.526	−8;4	−1	0.853	−8;7
Gender main effect		7	0.283	−6;19	7	0.348	−7;21	−8	0.015	−13;−2	**−11**	**0.005**	**−18;−3**
Cohort-by-gender interaction		20	0.120	−5;44	14	0.341	−14;42	3	0.606	−9;15	8	0.307	−7;23
Decomposed interaction (simple) effects													
Cohort	Pandemic (vs. non-pandemic) among boys	−12	0.204	−30;6	1	0.960	−20;21	−4	0.419	−13;5	−5	0.379	−16;6
	Pandemic (vs. non-pandemic) among girls	8	0.356	−9;25	14	0.143	−5;33	−1	0.909	−9;8	3	0.582	−7;13
Gender	Girls (vs. boys) in pandemic cohort	15	0.066	−1;32	13	0.182	−6;32	−6	0.142	−14;2	−7	0.159	−17;3
	Girls (vs. boys) in non-pandemic cohort	−4	0.650	−22;14	−1	0.942	−21;20	−9	0.045	0;18	**−15**	**0.008**	**−26;−4**

Linear models used for all variables. Linear regressions (instead of mixed-effect models) were used for model 2 because we could not collect accelerometer data in grade 7 for the pandemic cohort. Bolded values indicate *p*≤0.01 (main and simple effects) or *p* < 0.1 (interaction effects)

*PA* Physical activity, *MVPA* Moderate-to-vigorous PA, *min* minutes

^a^Reference groups for the analyses were: boys, grade 7, and pandemic cohort

b = unstandardized regression coefficients

**Table 6:** Effects of school transition, pandemic, and gender on accelerometry-measured sedentary and sleep time

		Sedentary time (hrs/weekdays)			Sedentary time (hrs/weekend days)			Meeting sleep guidelines on weekdays (vs. not)			Meeting sleep guidelines on weekend (vs. not)		
		b	*p*	95%CI	b	*p*	95%CI	OR	*p*	95%CI	OR	*p*	95%CI
Model 1: Mixed-effect linear and logistic regression models testing grade and gender main effects and their interaction in the non-pandemic cohort ^a^													
Grade main effect		**0.93**	**0.000**	**0.61;1.25**	**0.41**	**0.007**	**0.11;0.72**	0.48	0.035	0.24;0.95	0.54	0.069	0.28;1.05
Gender main effect		0.34	0.036	0.02;0.66	0.46	0.024	0.06;0.85	0.64	0.143	0.35;1.16	**0.39**	**0.010**	**0.19;0.80**
Grade-by-gender interaction		−0.02	0.940	−0.66;0.62	−0.28	0.357	−0.88;0.32	**0.20**	**0.029**	**0.05;0.85**	0.69	0.568	0.19;2.48
Decomposed interaction (simple) effects													
Grade	Grade 8 (vs. 7) among boys	**0.94**	**0.000**	**0.50;1.38**	0.56	0.011	0.13;0.99	0.91	0.830	0.40;2.08	0.64	0.300	0.27;1.49
	Grade 8 (vs. 7) among girls	**0.91**	**0.000**	**0.45;1.38**	0.28	0.197	−0.14;0.70	**0.19**	**0.006**	**0.06;0.61**	0.44	0.105	0.16;1.19
Gender	Girls (vs. boys) in grade 7	0.35	0.080	−0.04;0.74	0.55	0.015	0.11;1.00	0.96	0.906	0.48;1.91	0.45	0.055	0.20;1.02
	Girls (vs. boys) in grade 8	0.32	0.224	−0.20;0.85	0.27	0.343	−0.29;0.83	0.20	0.012	0.06;0.69	0.31	0.038	0.10;0.94
Model 2: Linear and logistic regression models testing cohort and gender main effects and their interaction in grade 8 students from both cohorts ^a^													
Cohort main effect		**−0.53**	**0.004**	**−0.90;−0.17**	−0.51	0.018	−0.93;−0.09	**2.89**	**0.001**	**1.55;5.39**	**2.39**	**0.002**	**1.40;4.20**
Gender main effect		0.02	0.903	−0.34;0.39	0.14	0.516	−0.28;0.55	**0.40**	**0.002**	**0.23;0.71**	0.68	0.155	0.40;1.16
Cohort-by-gender interaction		−0.53	0.158	−1.27;0.21	−0.36	0.409	−1.21;0.50	2.26	0.244	0.57;8.90	2.58	0.109	0.81;8.24
Decomposed interaction (simple) effects													
Cohort	Pandemic (vs. non-pandemic) among boys	−0.28	0.288	−0.79;0.23	−0.31	0.327	−0.94;0.31	2.16	0.052	0.99;4.69	1.54	0.269	0.72;3.32
	Pandemic (vs. non-pandemic) among girls	**−0.81**	**0.003**	**−1.34;−0.29**	−0.67	0.022	−1.25;−0.10	**4.88**	**0.006**	**1.58;15.08**	**3.98**	**0.002**	**1.66;9.52**
Gender	Girls (vs. boys) in pandemic cohort	−0.23	0.378	−0.73;0.28	−0.01	0.967	−0.55;0.53	0.50	0.040	0.26;1.00	0.94	0.841	0.49;1.80
	Girls (vs. boys) in non-pandemic cohort	0.31	0.263	−0.23;0.85	0.35	0.294	−0.30;1.00	0.22	0.013	0.07;0.73	0.36	0.037	0.14;0.94

Linear (sedentary time variables) and logistic (sleep variables) regressions, instead of mixed-effect models, were used for model 2 because we could not collect accelerometer data in grade 7 for the pandemic cohort. Bolded values indicate *p*≤0.01 (main and simple effects) or *p* < 0.1 (interaction effects)

*OR* Odd ratios, *min* minutes, *hrs* hours

^a^Reference groups for the analyses were: not meeting sleep recommendations, boys, grade 7, and pandemic cohort

b = unstandardized regression coefficients

## Discussion

This study is among the few [19, 20] to prospectively examine how transitioning to secondary school affects obesity-related behaviors in children, considering gender and pandemic conditions. Overall, the transition to secondary school was associated with declines in several lifestyle behaviors, including reduced fruit and vegetables intake, light PA, MVPA, and the proportion of children meeting sleep duration recommendations, as well as increases in sedentary time and screen time. However, some of these effects varied significantly by gender and cohort. For example, girls consistently had lower odds of meeting sleep recommendations and lower engagement in MVPA than boys, while the pandemic cohort showed decreases in sedentary time and screen use, and increases in weekday fruit and vegetables intake. The substantial changes in behaviors observed during the transition highlight the need for interventions aimed at mitigating declines in health behaviors and supporting healthy lifestyle habits. Gender-specific strategies may be particularly important for addressing disparities in sleep and MVPA patterns. Moreover, while the pandemic cohort showed improvements in eating habits and reduced sedentary time during the transition, elevated screen use was also observed and may warrant targeted intervention to address potential long-term impacts that could persist into adulthood.

In alignment with previous studies [19], recreational screen time increased during the transition to secondary school in the non-pandemic cohort, whereas screen-watching time (e.g., TV, movies, sports) remained stable. Although this may appear contradictory, it likely reflects differences in how parents monitor various types of screen use. Prior research has shown that decreases in supportive media parenting practices during this transition are associated with greater total screen time, potentially because parents relax certain rules as children gain autonomy [18]. In our study, stability in traditional screen watching may indicate that such rules remained in place for that medium, while increases in personal device use (e.g., phones or tablets) contributed to the rise in total screen time. To address this trend, future research should explore parenting strategies and school policies aimed at mitigating increases in recreational screen time during this transitional period.

Under pandemic conditions, recreational screen-time decreased with the transition, likely due to increased screen access for remote schooling and social interactions [47]. Previous studies found parents were more lenient with screen time at the onset of the pandemic, as they adapted to new working conditions and familial routines [47]. Although the pandemic cohort reduced recreational screen time in grade 8 after returning to in-person schooling, screen time levels did not return to pre-pandemic norms, suggesting difficulty in reversing established permissive patterns [48]. Notably, the pandemic cohort experienced a greater increase in screen-watching activities during the transition. This trend may reflect the increasing integration of screens into parenting routines, as parents navigate the challenges of balancing work and family responsibilities [48]. A qualitative study [47] conducted during the pandemic noted that some families consciously chose not to return to pre-pandemic busy schedules, which may lead to more opportunities for screen watching as a family, as observed in this study.

The transition also led to a decline in the proportion of girls meeting sleep recommendations (especially on weekdays), consistent with other studies highlighting gender differences in sleep patterns [49]. Early maturation has been a common explanation for poorer sleep in female adolescents. However, recent research suggests that higher anxiety levels in girls may be a more significant factor [48], and girls in our sample have shown greater anxiety scores than boys [50]. Notably, gender differences in sleep patterns were less pronounced on weekends (and inconsistent by measurement methods), though pandemic girls (as opposed to boys) showed a downward trend from grades 7 to 8. This decline may reflect shifts in familial routines, as studies have noted that family priorities shifted, leading to fewer weekend activities for children [46] and possibly making parents less strict about sleep schedules.

The study revealed shifts in PA among children transitioning to secondary schools, where both light PA and MVPA decreased and sedentary time increased. Notably, some inconsistencies were observed between assessment methods for MVPA. Accelerometry data showed a sharper decline in daily minutes of MVPA among boys, while self-reports illustrate that girls exhibited fewer days per week with at least 60 min of MVPA. This discrepancy, noted by others [20], suggests that while some children may consistently achieve 60 min of MVPA on certain days, their total activity can still decrease significantly, highlighting accelerometry as a more accurate measure of activity duration than self-reports. However, the gendered differences in PA align with other studies showing that fewer girls meet PA recommendations than boys, especially through adolescence [13]. This differentiation in MVPA likely emerged before the transition, as girls’ MVPA measured via accelerometry was already lower since elementary school. While the transition is often considered a trigger for changes in PA, including a decrease in MVPA and light PA and an increase in sedentary time [19], our findings indicate that gender differences in MVPA are influenced by additional factors that may occur earlier in life. Given the observed declines, maintaining girls’ engagement PA and sports is crucial. Future research should explore whether these differences are explained by unmet needs, social norms, biological maturation, and/or limited opportunities.

While gender differences in MVPA persisted under pandemic conditions, no pandemic-related effects were observed for light PA or MVPA in secondary school. However, sedentary time was lower post-transition during the pandemic. Although some early-pandemic studies reported declines in PA [22], longitudinal research shows a return to pre-pandemic levels over time [21]. This may reflect a shift toward more outdoor independent activities during the pandemic. In BC, public health officials encouraged outdoor social interaction, which likely prompted parents to send their children outside more frequently, potentially benefiting all children, and especially girls, who have traditionally had less outdoor independent mobility than boys [51].

Dietary behaviors shifted during the transition to secondary school. We observed a small but significant decline in weekday dietary quality among boys in the non-pandemic cohort, along with a decrease in fruit and vegetable intake across both genders. Adolescence is frequently associated with deteriorating dietary habits [7, 14, 15], and our findings support a modest decline during this transitional period. Notably, baseline intake of fruits and vegetables was already low. As children gain greater autonomy over their food choices (such as preparing their own lunches or purchasing snacks), it is important to equip them with the skills and support needed to make healthy decisions. Establishing healthy eating habits at a younger age may help ease this adjustment and promote better long-term dietary behaviors.

Interestingly, the pandemic may have led to modest improvements in certain dietary behavior, specifically in weekday fruit and vegetables intake. This finding aligns with other studies reporting positive changes in some dietary components during the pandemic [7, 14, 15]. Remote learning likely provided more opportunities to eat at home and greater access to food throughout the day, leading to more frequent family meals [48]. This may help explain the rise in weekday fruit and vegetable consumption observed in the pandemic cohort, particularly among children with access to higher-quality foods in the home environment.

### Limitations and strengths

This study’s findings should be interpreted in light of several limitations. First, reliance on self-reported data, though collected using validated tools, may introduce measurement error that could obscure true behavioral changes. Second, while demographic characteristics were similar across grades, attrition in prospective studies may affect the results, particularly if children from disadvantaged households are underrepresented post-transition. Third, the study was conducted in BC, Canada, which may limit generalizability to other regions or countries, especially those that implemented different pandemic-related policies. Fourth, although the naturalistic design allowed for comparison between pandemic and non-pandemic conditions, differences between cohorts cannot be definitively attributed to the pandemic itself. Moreover, as this was an observational study, longitudinal changes observed from grade 7 to 8 cannot be solely attributed to the transition to secondary school; they may also reflect age-related developmental changes or other social and environmental factors. Future research could benefit from examining a broader set of variables that contribute to behavioral changes during this period, helping to clarify underlying mechanisms and inform targeted intervention strategies. A key strength of the study, however, is that all families in the pandemic cohort were recruited prior to the onset of the pandemic, ensuring comparable socio-demographic characteristics between the two cohorts.

## Conclusions

In conclusion, changes in children’s screen time, sleep, sedentary behavior, PA, and dietary habits were observed during the transition to secondary school, with patterns varying by gender and level of pandemic exposure. Many gender differences were either present prior to the transition or emerged during this period, but the extent to which these changes were triggered by the transition requires further investigation, as they may also reflect other developmental or environmental factors. These findings underscore the need for targeted interventions to support the maintenance of healthy behaviors during this critical stage of life. Designing effective health promotion strategies will require a better understanding of the factors driving divergent behavioral patterns between boys and girls during the transition. Furthermore, as the pandemic likely disrupted children’s behavioral trajectories (particularly in relation to screen use), ongoing support may be needed to help reset these patterns as children move toward adulthood.

## Data Availability

The data used in this study are not publicly available. Data are however available upon reasonable request to the corresponding author, and with permission of the Research Ethics Board at the University of British Columbia.
